# Contemporary Diagnostic and Therapeutic Approaches in Endometriosis: A Systematic Review

**DOI:** 10.7759/cureus.106354

**Published:** 2026-04-03

**Authors:** Snigdha Tanaya Nayak, Saumya, Nikeeta Ashokrao Khanorkar, Shreya Vijayasankar, Aswin Bhikhalal Ghori, Satyanarayana Kummari

**Affiliations:** 1 Department of Radiodiagnosis, DRIEMS Institute of Health Sciences &amp; Hospital, Tangi, IND; 2 Department of Obstetrics and Gynaecology, Government Medical College, Satna, Satna, IND; 3 Department of Obstetrics and Gynaecology, Government Medical College Nagpur, Nagpur, IND; 4 Department of Obstetrics and Gynaecology, Maharashtra University of Health Sciences, Nashik, IND; 5 Department of Obstetrics and Gynaecology, The Tamil Nadu Dr. M.G.R. Medical University, Chennai, IND; 6 Department of Obstetrics and Gynaecology, J.Z.M and N General Hospital, Nadiad, IND; 7 Department of Radiodiagnosis, All India Institute of Medical Sciences, Bibinagar, Bibinagar, IND

**Keywords:** biomarkers, deep infiltrating endometriosis, diagnostic approaches, digital therapeutics, endometriosis, microrna, systematic review, transvaginal sonography

## Abstract

Endometriosis is a chronic inflammatory disorder characterized by pelvic pain, infertility, and reduced quality of life, and is frequently associated with prolonged diagnostic delay and variable treatment response. Although advances in noninvasive diagnostics and multimodal therapies have accelerated in recent years, the evidence remains heterogeneous and insufficiently integrated into clinical pathways. This systematic review provides a qualitative synthesis of contemporary (2015-2025) diagnostic and therapeutic evidence in endometriosis rather than a comparative effectiveness analysis. A structured search of major electronic databases (e.g., PubMed, Scopus, and Web of Science) following Preferred Reporting Items for Systematic Reviews and Meta-Analyses (PRISMA)-based study selection methods identified eligible diagnostic accuracy studies, biomarker validation cohorts, randomized controlled trials, and non-randomized interventional studies. Ten studies met the inclusion criteria, comprising four diagnostic and six therapeutic investigations, with conclusions limited by heterogeneity and the modest volume of available evidence, particularly for therapeutic interventions. Diagnostic findings indicated that transvaginal sonography demonstrates site-dependent performance for deep infiltrating disease, while serum microRNA models combined with machine-learning classifiers achieved high discriminatory accuracy in surgically confirmed cohorts, which may limit generalizability. Urinary microRNA signatures show slightly lower but comparable performance. Therapeutic evidence encompassed pharmacologic, nutraceutical, digital, exercise-based, and behavioral interventions. Melatonin demonstrated improvement in sleep-related outcomes, but these symptom-specific benefits did not consistently translate into broader therapeutic effectiveness, with inconsistent effects on pain. Omega-3 supplementation did not significantly reduce pain, and digital or behavioral modalities, including immersive virtual reality and mindfulness-based interventions, produced short-term reductions in pain intensity or improvements in quality-of-life domains, with pilot data indicating feasibility despite limited evidence of sustained efficacy, supporting their potential for evaluation in larger trials. Contemporary research reflected meaningful innovation but remained constrained by small sample sizes, short follow-up, surgical cohort bias in diagnostic studies, and heterogeneity in outcome reporting. Robust multicenter validation, standardized endpoints, and phenotype-informed therapeutic trials are required to translate emerging innovations into sustained clinical benefit.

## Introduction and background

Endometriosis is a chronic, estrogen-dependent inflammatory disease characterized by the proliferation of endometrial-like tissue outside the uterine cavity [1]. It is a disease affecting women of reproductive age and is commonly associated with dysmenorrhea, chrthe onic pelvic pain, dyspareunia, and infertility [2]. The concept of endometriosis as a systemic disease with immune dysregulation, neuroinflammation, and central sensitization, a mechanism of amplified pain perception due to altered neural processing, as contributing factors, has been increasingly recognized, contributing to persistent pain and overall symptom burden [1]. The disease has been reported to result in substantial reductions in quality of life, daily dysfunction, and increased healthcare utilization. The contemporary paradigm of care places increasing emphasis on patient-centered and multimodal approaches that integrate diagnostic precision with individualized therapeutic strategies [3].

There remains a critical challenge in achieving timely and accurate diagnosis despite its clinical impact. Laparoscopic visualization, with or without histopathologic confirmation, is the reference standard for diagnosis, and this distinction was considered when interpreting diagnostic accuracy, due to its influence on reference standard validity. However, reliance on surgical confirmation contributes to diagnostic delay and under-recognition, particularly in patients with nonclassical presentations [4]. The limited sensitivity of conventional imaging modalities for superficial peritoneal disease has also highlighted the historical reliance on invasive assessment. Advances in transvaginal sonography have improved the detection of deep infiltrating endometriosis, a severe form of disease defined by lesions infiltrating more than 5 mm into pelvic structures, as well as parametrial involvement, although diagnostic accuracy remains dependent on lesion location and operator expertise [5,6]. These limitations underscore the importance of developing reliable noninvasive diagnostic tools to enable earlier diagnosis and stratified management.

This has placed molecular biomarker research at the forefront. Circulating microRNAs, small non-coding RNA molecules used as potential circulating biomarkers, have demonstrated discriminatory ability between individuals with endometriosis and control populations [7,8]. With advances in bioengineering and precision medicine, molecular profiling is increasingly being integrated with imaging data to improve diagnostic performance [9,10]. However, biomarker studies remain heterogeneous in terms of cohort selection, assay techniques, and analytical methods, and often lack external validation. Inconsistencies in reporting standards and reference definitions further limit clinical translation [10]. This necessitates the systematic synthesis of diagnostic performance and generalizability across studies, alongside separate evaluation of therapeutic effectiveness across heterogeneous intervention strategies, to critically interpret the available evidence.

Therapeutic approaches have also evolved in response to the complexity and chronicity of the disease. Hormonal suppression remains a cornerstone of management but may be ineffective in some patients due to recurrence, adverse effects, and incomplete pain control. Recent literature emphasizes individualized treatment strategies that consider both deep infiltrating bladder endometriosis (DUBT) and non-urinary bladder endometriosis (Non-UBT) [11,12]. Alternative therapies have also gained attention, including melatonin, which has been investigated for its analgesic and sleep-regulating properties. There are conflicting results from randomized trials, with some showing no significant decrease in pelvic pain and others demonstrating improvements in sleep-related outcomes [13,14]. Nutraceutical interventions, such as omega-3 polyunsaturated fatty acids, reflect growing interest in anti-inflammatory strategies, although current randomized evidence does not demonstrate clear superiority over placebo in pain reduction [15,16].

Parallel to this, there is increasing recognition of the role of multimodal and non-pharmacologic interventions. Holistic care models integrate medical treatment with lifestyle modification, psychological support, and symptom-targeted management of pain and functional impairment, acknowledging that disease burden is not solely determined by lesion extent [17,18]. Digital therapeutic approaches, such as immersive virtual reality platforms, have demonstrated short-term reductions in pain intensity in randomized controlled trials and may serve as adjunctive tools for symptom modulation [19]. Behavioral interventions, including mindfulness-based therapies, further highlight the importance of improving quality-of-life outcomes alongside pain control.

Despite growing research activity, the evidence base remains fragmented. Diagnostic studies vary in reference standards and performance metrics, while therapeutic trials differ in intervention types, outcome measures, and follow-up durations, limiting direct comparison and integration into unified clinical pathways. This systematic review therefore provides a broad qualitative synthesis of both diagnostic and therapeutic evidence rather than a focused comparative evaluation of a single clinical pathway, encompassing advances in noninvasive imaging, molecular biomarkers, pharmacologic and non-pharmacologic interventions, while identifying methodological limitations and evidence gaps to inform future research and clinical practice.

## Review

Methods

Study Design and Reporting

The purpose of this systematic review was to synthesize contemporary diagnostic and therapeutic approaches in endometriosis. The review was designed and reported in accordance with the Preferred Reporting Items for Systematic Reviews and Meta-Analyses (PRISMA) guidelines [20]. Study screening and full-text selection were performed independently by multiple reviewers, with disagreements resolved through discussion or consultation with a third reviewer.

Information Source and Search Strategy

A structured electronic search was conducted in PubMed/MEDLINE, Scopus, Web of Science, and Embase to identify studies published over 10 years (January 2015 to December 2025). The search strategy combined controlled vocabulary (e.g., MeSH and Emtree terms) and free-text terms related to endometriosis, diagnostic approaches, and therapeutic interventions. Diagnostic-related terms included transvaginal sonography, biomarkers, microRNA, and machine learning-based classification. Therapeutic terms included pharmacologic agents, melatonin, omega-3 supplementation, virtual reality, telehealth, exercise interventions, and mindfulness-based programs. Boolean operators (AND, OR) and database-specific filters were applied as appropriate, including language (English) and publication date (2015-2025) restrictions across all databases.

The full PubMed/MEDLINE search strategy was: (“endometriosis”[MeSH Terms] OR “endometriosis”) AND (“transvaginal sonography” OR “ultrasound” OR “biomarkers” OR “microRNA” OR “machine learning” OR “melatonin” OR “omega-3” OR “virtual reality” OR “exercise” OR “mindfulness”) AND (2015:2025[pdat]) AND (English[lang]).

Eligibility criteria

Inclusion criteria: Studies were included if they were published in English between January 2015 and December 2025 and identified across all searched databases (PubMed/MEDLINE, Scopus, Web of Science, and Embase). Eligible studies involved participants with suspected or confirmed endometriosis, including both surgical and clinically diagnosed cohorts. Studies were required to evaluate either diagnostic methods (e.g., imaging modalities, molecular biomarkers such as microRNA profiling, or machine-learning models) or therapeutic interventions (e.g., pharmacologic, nutraceutical, digital, exercise-based, or behavioral approaches). Eligible study designs included randomized controlled trials (including pilot RCTs), diagnostic accuracy studies, diagnostic validation studies, biomarker discovery cohorts, and non-randomized before-after studies. Studies were required to report extractable quantitative outcomes, including diagnostic performance measures (e.g., sensitivity, specificity, area under the receiver operating characteristic curve (ROC-AUC)) or therapeutic outcomes (e.g., pain intensity, sleep quality, feasibility, or quality-of-life measures).

Exclusion criteria: Studies were excluded if they were published in a non-English language, before 2015 or after 2025, did not focus on endometriosis diagnosis or treatment, or did not provide extractable outcome data. Full-text articles that did not meet the predefined eligibility criteria were excluded following detailed assessment.

Study Selection

The database search yielded a total of 252 records after removal of duplicates. Following title and abstract screening of 211 records, 164 were excluded based on irrelevance. Full-text assessment was conducted for 47 articles, of which 37 were excluded due to insufficient outcome data, non-English publication, or failure to meet inclusion criteria. A total of 10 studies were included in the qualitative synthesis.

Data Extraction

Data were extracted using a predefined and standardized template. Data extraction was performed independently by multiple reviewers, with discrepancies resolved through discussion or consultation with a third reviewer. Extracted variables included author and year, country, study design, sample size, participant characteristics, study domain (diagnostic or therapeutic), index test or intervention, comparator or reference standard, follow-up duration, primary outcomes, and key findings.

For diagnostic studies, extracted outcomes included sensitivity, specificity, and ROC-AUC, along with the reference standard (typically laparoscopy with or without histologic confirmation). For therapeutic studies, extracted outcomes included pain intensity measures (e.g., visual analogue scale or numerical rating scale), sleep quality indices (e.g., Pittsburgh Sleep Quality Index), feasibility outcomes, and quality-of-life measures (e.g., Endometriosis Health Profile domains).

Risk of Bias Assessment

Risk of bias was assessed using design-specific appraisal tools. Randomized controlled trials were evaluated using the Cochrane Risk of Bias 2 (RoB 2) tool [16], which assesses bias across domains including randomization, deviations from intended interventions, missing outcome data, outcome measurement, and selective reporting.

Non-randomized before-after studies were assessed using domains adapted from the Risk Of Bias In Non-Randomized Studies - of Interventions (ROBINS-I) framework [6], including confounding, participant selection, intervention classification, deviations from intended interventions, missing data, outcome measurement, and reporting bias.

Diagnostic accuracy and biomarker studies were assessed using the quality assessment of comparative diagnostic accuracy studies (QUADAS-2) tool [9], covering domains of patient selection, index test performance and interpretation, reference standard, and flow and timing. Domain-level judgments were synthesized into overall risk-of-bias categories (low, some concerns, or high) based on the highest level of bias identified.

Data Synthesis

A qualitative narrative synthesis was conducted due to substantial heterogeneity across studies. Variability in diagnostic modalities (imaging, serum, and urinary biomarkers), intervention types (pharmacologic, nutraceutical, digital, exercise-based, and behavioral), outcome measures, and follow-up durations precluded meta-analysis. Diagnostic and therapeutic evidence were synthesized separately to maintain methodological coherence. Interpretation of findings incorporated risk-of-bias assessments, study design, sample size, and consistency of reported outcomes to contextualize the strength and limitations of the evidence base, with greater emphasis placed on higher-quality studies and those with lower risk of bias.

Results

Study Selection

**Figure 1:**
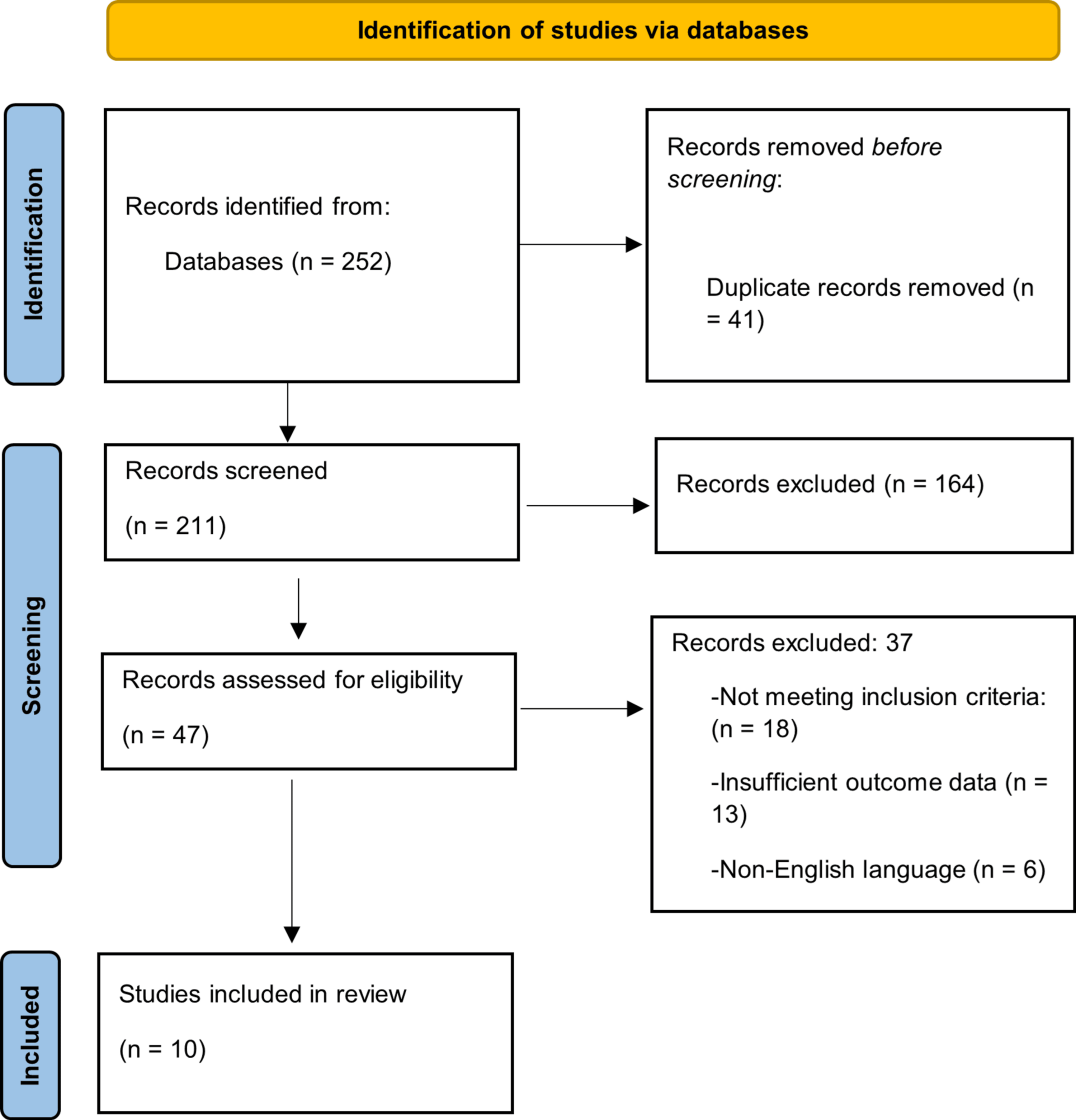
PRISMA flow diagram PRISMA: Preferred Reporting Items for Systematic Reviews and Meta-Analyses.

The PubMed search (2015-2025) identified 252 records. Following removal of 41 duplicates, 211 records underwent title and abstract screening, during which 164 records were excluded for irrelevance to endometriosis diagnosis or treatment. Full-text eligibility assessment was subsequently performed for 47 articles. Of these, 37 were excluded due to insufficient extractable outcome data, publication in a non-English language, or failure to meet the predefined inclusion criteria. Ultimately, 10 studies met eligibility requirements and were included in the qualitative synthesis, and given the small and heterogeneous evidence base, findings were interpreted with caution to avoid overgeneralization. The study selection process is presented in Figure 1 using a PRISMA flow diagram.

Characteristics of Included Studies

The 10 studies included were four diagnostic studies and six therapeutic studies, which were synthesized separately to maintain methodological clarity between diagnostic and therapeutic evidence streams. Designs of study were randomized controlled trials, pilot randomized controlled trials, diagnostic accuracy studies, diagnostic validation studies, a biomarker discovery cohort, and one mixed-method before-and-after study.

Diagnostic trials evaluated imaging, molecular biomarker-based evaluation strategies, such as microRNA profiling, on a case-control study of surgery using laparoscopy with or without histologic validation as reference. The pharmacologic agents, nutraceutical interventions, immersive digital therapeutics, exercise-based programs provided either through virtual reality or telehealth, and a mindfulness-based behavioral intervention were assessed with therapeutic studies. The sample sizes were small pilot cohorts and large diagnostic cohorts. The duration of follow-up ranged between acute short-term assessment and formal intervention of up to eight weeks and two months.

Table 1 provides detailed features of the included studies, including justification for the inclusion of studies such as Kupec et al. [21], given the clinical and pathophysiological overlap between adenomyosis and endometriosis.

**Table 1:** Key study characteristics and findings Studies with overlapping gynecologic conditions (e.g., adenomyosis) were included due to shared symptomatology and pathophysiological mechanisms relevant to endometriosis. TVS: Transvaginal sonography; miRNA: MicroRNA; RNA-seq: RNA sequencing; qRT-PCR: Quantitative reverse transcription polymerase chain reaction; ROC-AUC: Receiver operating characteristic–area under the curve; NRS: Numerical rating scale, PSQI: Pittsburgh Sleep Quality Index; PUFA: Polyunsaturated fatty acids; BPI: Brief Pain Inventory; RCT: Randomized controlled trial; VR: Virtual reality; VAS: Visual analogue scale; MBSR: Mindfulness-based stress reduction; EHP: Endometriosis Health Profile; ML: Machine learning; DIE: Deep infiltrating endometriosis; FDR: False discovery rate.

Study	Study design	Sample size	Population	Domain	Diagnostic test/ intervention	Comparator/ Reference standard	Follow-up	Primary outcome	Key findings
Garzon et al., 2024 [5]	Diagnostic accuracy study	198	Women suspected of deep infiltrating endometriosis	Diagnostic	Transvaginal sonography (TVS)	Surgery + histopathology	Preoperative	Sensitivity/specificity	High diagnostic accuracy for uterosacral ligament and intestinal DIE; variable by lesion site
Vash-Margita et al., 2025 [7]	Prospective cohort	63 recruited; 51 analyzed (31 endometriosis, 20 controls)	Adolescents/young adults undergoing gynecologic surgery	Diagnostic	Serum miRNA RNA-seq	Surgical diagnosis	Cross-sectional	Differential miRNA expression (DESeq2, FDR <0.05)	859 miRNAs differentially expressed; adolescent-specific molecular signature identified
Moustafa et al., 2020 [8]	Diagnostic validation study	100 (41 endometriosis; 59 controls)	Women undergoing laparoscopy	Diagnostic	Serum miRNA qRT-PCR + Random Forest	Surgical + histologic confirmation	Cross-sectional	ROC-AUC	Random Forest AUC = 0.939 (independent validation set)
Söderman et al., 2023 [13]	Double-blind randomized placebo-controlled trial	40	Women 18–50 with endometriosis + severe dysmenorrhea	Therapeutic	Melatonin 20 mg nightly	Placebo	2 cycles	Pelvic pain (NRS)	No significant difference vs placebo (p=0.45)
Esmaeilzadeh et al., 2025 [14]	Triple-blind randomized placebo-controlled trial	80 (40 melatonin; 40 placebo)	Infertile women with laparoscopic endometriosis + sleep disturbance	Therapeutic	Melatonin 5 mg daily (prolonged-release)	Placebo	2 months	PSQI change	Significant PSQI improvement vs placebo (p<0.001; η²=0.20; Cohen’s d=1); mean PSQI change −1.7
Abokhrais et al., 2020 [15]	Double-blind randomized pilot RCT	33 randomized (17 omega-3; 16 olive oil)	Women 18–50 with laparoscopic endometriosis + pelvic pain	Therapeutic	Omega-3 PUFA 1000 mg twice daily	Olive oil placebo	8 weeks	Feasibility + pain (BPI)	No significant between-group difference; feasibility confirmed
Merlot et al., 2022 [17]	Randomized controlled comparative study	45 randomized; 44 completed	Women with moderate-to-severe endometriosis pelvic pain	Therapeutic	Endocare (20-min VR digital therapeutic)	Digital tablet control	240 minutes (T0–T240)	Mean pain intensity at 60 min	Significant pain reduction in the VR group; moderate clinical effect (~30%)
Lutfi et al., 2023 [18]	Randomized controlled 3-arm pilot	22 randomized (VR=8; telehealth=8; control=6)	Women 18–45 with endometriosis pelvic pain	Therapeutic	Single-session VR-delivered exercise	Telehealth exercise; control	Acute (baseline–48h)	VAS pelvic pain	No between-group difference (p=0.45); η²=0.10 interaction effect; smaller pain increase in VR/telehealth vs control
Miazga et al., 2022 [19]	Mixed-methods before–and–after study	15	Patients with clinical or surgical endometriosis	Therapeutic	Virtual Mindfulness-Based Stress Reduction (MBSR)	Baseline comparison	Post-intervention	Endometriosis Health Profile (EHP) domains	Significant improvement in multiple QoL domains; no change in pain or medication use
Kupec et al., 2025 [21]	Diagnostic biomarker study with ML	52 (34 endometriosis; 18 controls)	Surgical candidates with suspected endometriosis, including cases with overlapping features of adenomyosis	Diagnostic	Urinary miRNA sequencing + ML classifiers	Laparoscopy ± histology	Preoperative	Diagnostic accuracy (ROC-AUC)	Random Forest AUC = 0.91; accuracy = 0.81

Contemporary Diagnostic Approaches

Diagnostic and therapeutic studies were synthesized separately due to differences in study design, outcome measures, and clinical objectives. There were four studies on diagnostic modalities. One imaging study evaluated the use of transvaginal sonography in deep infiltrating endometriosis [5]. There was variation in sensitivity and specificity according to the anatomical site, with the uterosacral ligament and intestinal lesions demonstrating the highest performance, highlighting that diagnostic accuracy is site-dependent and should be interpreted accordingly in clinical decision-making. The reference standard was laparoscopy with or without histopathology.

**Figure 2:**
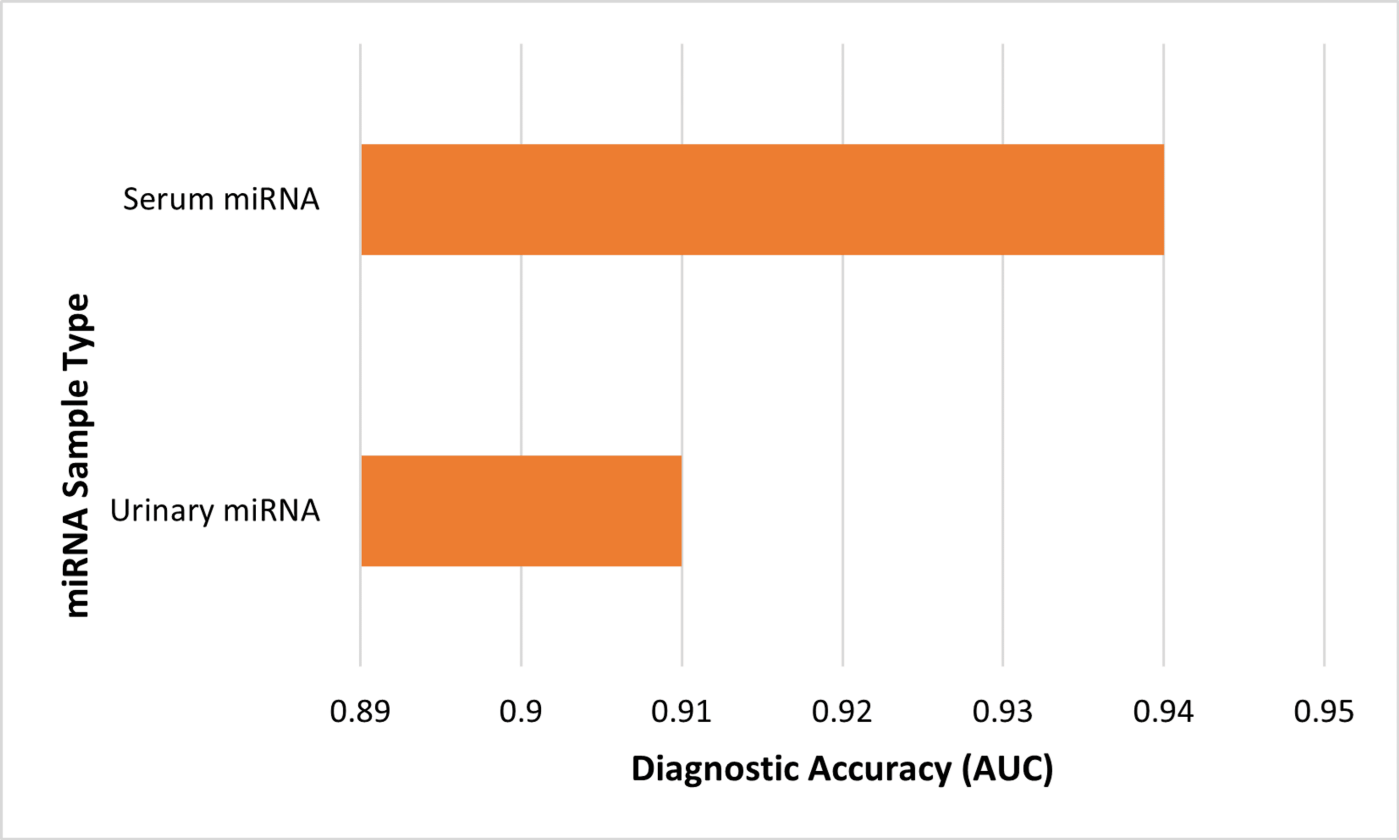
Serum vs urinary miRNA diagnostic accuracy (AUC) AUC: Area under the curve. Derived from [8,21].

Three studies evaluated microRNA-based biomarkers. Serum microRNA profiling demonstrated strong discriminatory performance, with a random forest ROC-AUC of 0.939 reported in an independent validation cohort [8]. Urinary microRNA profiling using machine learning yielded a classification accuracy of 0.81 [21]. A biomarker discovery cohort identified 859 differentially expressed miRNAs with an adolescent-specific molecular signature [7]. However, as all diagnostic studies were conducted in surgically confirmed cohorts, these performance estimates may be inflated and are not fully generalizable to broader clinical populations, warranting cautious interpretation. Figure 2 presents the comparative ROC-AUC values.

Contemporary Therapeutic Approaches

Therapeutic evidence was analyzed independently to account for heterogeneity in intervention types and outcome reporting. Six studies were included for therapeutic evaluation, encompassing pharmacologic, nutraceutical, digital, exercise-based, and behavioral interventions. Two randomized placebo-controlled trials examined melatonin. In the higher-dose trial (20 mg nightly), there was no statistically significant reduction in pelvic pain compared with placebo (p=0.45) [13]. In contrast, the lower-dose prolonged-release trial (5 mg daily) demonstrated a statistically significant improvement in sleep quality compared with placebo (p<0.001), with an effect size of 0.20 and a Cohen’s d of 1.0 [14]; however, no significant improvement in pain outcomes was observed [14].

One double-blind pilot randomized controlled trial evaluated omega-3 polyunsaturated fatty acids (1000 mg twice daily) over eight weeks, with no statistically significant difference in pain outcomes observed between intervention and placebo groups, although feasibility outcomes were successfully achieved [15].

Technology-based interventions included immersive virtual reality and a pilot three-arm randomized trial comparing VR-delivered exercise, telehealth-delivered exercise, and control. The immersive virtual reality intervention demonstrated a statistically significant reduction in pain intensity at 60 minutes compared with a digital tablet control, with a mean difference of approximately 30, indicating short-term symptom modulation rather than sustained therapeutic benefit [17]. In the virtual reality/telehealth exercise pilot trial, no statistically significant differences in pain scores were observed between groups (p=0.45); however, a group-by-time interaction effect (η² = 0.10) suggested reduced pain escalation in the intervention groups [18].

A mixed-methods before-after study evaluating a virtual mindfulness-based stress reduction (MBSR) program demonstrated statistically significant improvements in multiple domains of the Endometriosis Health Profile at post-intervention [19]. However, the absence of a control group indicates that these findings should be considered preliminary [19]. No statistically significant changes in pain scores or medication use were observed [19]. Overall, therapeutic evidence was limited by heterogeneity in intervention modalities, outcome measures, and follow-up durations, precluding quantitative meta-analysis.

Risk of Bias Assessment

In randomized controlled trials, the overall risk of bias was mainly low, with appropriate randomization, blinding, and use of comparators reported. Two pilot randomized controlled trials were of moderate risk due to small sample sizes and possible performance bias, and these risk-of-bias assessments were considered when interpreting the strength and reliability of the overall conclusions [16].

The diagnostic studies were rated as having a moderate risk of bias, most commonly due to patient selection and the use of surgically confirmed cohorts [9]. The non-randomized before-after study also demonstrated a moderate risk, primarily due to potential confounding and the absence of a control group [6]. Table 2 provides a summary of these domain-level judgments.

**Table 2:** Risk of bias assessment of the included studies

Study	Study design	Selection bias	Performance bias	Detection bias	Overall risk of bias
Garzon et al., 2024 [5]	Diagnostic accuracy study (TVS)	Moderate	Low	Low	Low
Vash-Margita et al., 2025 [7]	Prospective diagnostic cohort (serum miRNA RNA-seq)	Moderate	Low	Moderate	Moderate
Moustafa et al., 2020 [8]	Diagnostic validation study	Moderate	Low	Low	Moderate
Söderman et al., 2023 [13]	Double-blind randomized controlled trial	Low	Low	Low	Low
Esmaeilzadeh et al., 2025 [14]	Triple-blind randomized controlled trial	Low	Low	Low	Low
Abokhrais et al., 2020 [15]	Double-blind pilot randomized controlled trial	Moderate	Low	Low	Moderate
Merlot et al., 2022 [17]	Randomized controlled trial (VR intervention)	Moderate	Moderate	Moderate	Moderate
Lutfi et al., 2023 [18]	Pilot randomized controlled trial (VR/telehealth)	Moderate	Moderate	Moderate	Moderate
Miazga et al., 2024 [19]	Before–after mixed-methods study	Moderate	Moderate	Moderate	Moderate
Kupec et al., 2025 [21]	Diagnostic biomarker study (ML-based)	Moderate	Low	Moderate	Moderate

Discussion

Recent research on endometriosis shows not only the development of diagnosis but also treatment, but the evidence base remains heterogeneous and does not have clinical coherence. Ten studies concerning the topic, published between 2015 and 2025, were found and synthesized in this review; four were diagnostic studies, and six were therapeutic trials. The findings indicate that noninvasive diagnostics and adjunctive management of symptoms show promising but still preliminary improvements, despite limitations in external validation, cohort representativeness, and long-term outcome assessment.

Transvaginal sonography is also gaining a presence within the diagnostic arena, particularly in deep infiltrating endometriosis involving the posterior compartment and parametrial regions, with diagnostic performance influenced by operator experience, which is critical for real-world implementation. Selectivity to the location of lesions was also selective to deep lesions, as has been demonstrated in the larger literature on the topic, which demonstrated greater sensitivity to deep lesions. The reviews will point out that the quality of imaging remains closely related to the experience of the operators, and without standardization of the procedures [22]. These findings contribute to the growing significance of sonography in preoperative mapping and clinical triage, emphasizing that sonography will scarcely be able to fully replace surgical confirmation in cases that have a diagnostically uncertain nature.

Another important field of innovation is molecular diagnostics, primarily the circulating microRNA profiling. The serum-based validation study in this review showed a high discriminatory performance of the machine learning model, with ROC-AUC values approaching 0.94, although these findings remain investigational pending broader external validation. Urinary microRNA profiling was also performed with a high performance, though a bit lower. These findings are correlated to the past syntheses that characterized microRNAs as the promising noninvasive biomarkers [23]. However, the fact that most of the diagnostic cohorts were surgically confirmed patients posed the risk of spectrum bias, which can exaggerate the performance estimates. Generalizability is also further hampered by the degree of variation in the lab platforms and the lack of external validation to a large extent. Although diagnostic models containing accurate diagnostic signatures indicate that molecular signatures are to be employed in combination with imaging and computational modelling [9], no universal guidelines on clinical implementation are common. Future research would consider multicentric validation in more general clinical groups, which would be in line with past recommendations that there is a need to reduce the delay in diagnosis [24].

In therapeutic studies, there is more divergence than an optimum convergence to one strategy. Hormonal inhibition is still considered the baseline, but adjunctive treatment is actively investigated to resolve the problem of persistent symptoms [11,12]. Symptom-domain specificity is demonstrated in the melatonin trials: a high dose of melatonin made no significant difference in alleviating pelvic pain, whereas a low dose of the prolonged-release melatonin enhanced the quality of sleep. This indicates the possible advantage of treating sleep instead of the nociceptive pathways directly [25]. The quality-of-life outcomes have been proven to improve in real-world hormonal regimens [26], but there is limited long-term comparative effectiveness data.

Nutraceutical methods, such as omega-3 supplementation, failed to prove better effects than placebo in reducing pain. Though it is biologically plausible by its anti-inflammatory effects, no randomized evidence has been demonstrated to demonstrate a meaningful analgesic effect [27]. Such wider reviews of holistic strategies have also emphasized methodological diversity and scarcity of high-quality trial data [28].

An emerging area of research is digital and behavioral interventions. Immersive virtual reality was led to short-term pain reduction, whereas the digitally presented exercise and mindfulness-based interventions mainly enhanced the domain of immediate symptom modulation or quality of life. These results are aligned with the need to revise the conceptualization of endometriosis as a systemic disease with central sensitization [29]. Digital therapeutics can thus act as an adjunct to the multimodal care models, playing the role of a neuromodulatory adjunct, but not a disease-modifying therapy [30,31].

A qualitative narrative synthesis was conducted due to substantial heterogeneity across studies, which precluded meta-analysis. Variability in diagnostic modalities, intervention types, outcome measures, and follow-up durations necessitated a separate synthesis of diagnostic and therapeutic evidence to maintain methodological coherence. Interpretation of findings incorporated risk of bias assessments, study design, sample size, and consistency of reported outcomes to contextualize the strength and limitations of the evidence base.

## Conclusions

In this systematic review, eligible diagnostic and therapeutic studies were synthesized to evaluate contemporary approaches to endometriosis management. Endometriosis is a chronic inflammatory disease associated with delayed diagnosis and variable treatment response. Diagnostic findings suggested promising, but still evolving, progress toward clinically applicable noninvasive tools. Transvaginal sonography demonstrated good performance in detecting deep infiltrating disease, and circulating microRNA-based models showed high discriminatory ability, although predominantly within surgically confirmed cohorts, which may limit generalizability. Therapeutic approaches extended beyond conventional hormonal suppression to include pharmacologic adjuncts, nutraceuticals, digital interventions, and behavioral strategies, with reported benefits largely domain-specific and often short-term, such as improved sleep quality with prolonged-release melatonin and transient pain reduction with immersive virtual reality. Across both diagnostic and therapeutic domains, the strength of evidence was constrained by methodological heterogeneity, small sample sizes, short follow-up durations, and limited external validation, precluding meta-analysis. Future research should prioritize multicenter validation in non-surgical populations, standardized outcome reporting, and adequately powered, phenotype-informed therapeutic trials to support the integration of precision diagnostics with multimodal, patient-centered care.
